# Long-term development of performance, physiological, and training characteristics in a world-class female biathlete

**DOI:** 10.3389/fspor.2023.1197793

**Published:** 2023-06-15

**Authors:** Guro Strøm Solli, Andrine Håstul Flom, Rune Kjøsen Talsnes

**Affiliations:** ^1^ Department of Sports Science and Physical Education, Nord University, Bodø, Norway; ^2^ Centre for Elite Sports Research, Department of Neuromedicine and Movement Science, Norwegian University of Science and Technology, Trondheim, Norway

**Keywords:** endurance training, female athlete, junior athlete, shooting, training quality, XC skiing

## Abstract

**Purpose:**

The purpose of this study is to investigate the long-term development of performance, physiological, and training characteristics in a world-class female biathlete, with emphasis on differences between junior and senior athlete seasons.

**Methods:**

The participant is a highly decorated female biathlete with 22 (10 gold) medals from international championships and 28 individual World Cup wins. Performance development (ages 17–33), physiological tests (ages 22–33), and day-to-day physical and shooting training (ages 17–33) were analyzed. Training data were systemized by endurance [low-intensity training (LIT), moderate-intensity training (MIT), and high-intensity training (HIT)], exercise mode, and strength training. Shooting training recorded for each session included the number of shots fired during rest, LIT, MIT, HIT, or competitions and time spent on dry fire training.

**Results:**

The annual volume of physical training (409–792 h·season^−1^) and number of shots fired (1,163–17,328 shots·season^−1^) increased from the age of 17 to 28 followed by a subsequent reduction in physical training (range 657–763 h·season^−1^) and shots fired (13,275–15,355 shots·season^−1^) during the seasons of peak performance at ages 31–33. Maximal oxygen uptake in roller ski skating increased by 10% (62.9–69.2 ml·kg^−1^·min^−1^) from the age of 22 to 27. The physical training volume was 48% higher (694 ± 60 vs. 468 ± 23 h·season^−1^, *P *= .030), with 175% more shots fired (14,537 ± 1,109 vs. 5,295 ± 3,425 shots·season^−1^, *P *= .016) as a senior athlete than a junior athlete. In the physical training, these differences were mainly explained by higher volumes of LIT (602 ± 56 vs. 392 ± 22 h·season^−1^, *P *= .032) and MIT (34 ± 1 vs. 7 ± 2 h·season^−1^, *P *= .001) but less HIT (27 ± 1 vs. 42 ± 3 h·season^−1^, *P *= .006) as a senior than a junior. In line with this, shooting training as a senior included more shots fired both at rest (5,035 ± 321 vs. 1,197 ± 518 shots·season^−1^, *P *= .011) and during LIT (7,440 ± 619 vs. 2,663 ± 1,975 shots·season^−1^, *P *= .031), while a smaller insignificant difference was observed in the number of shots fired in connection with MIT, HIT, and competitions (2,061 ± 174 vs. 1,435 ± 893 shots·season^−1^, *P *= .149).

**Conclusions:**

This study provides unique insights into the long-term development of physical and shooting training from junior to senior in a world-class female biathlete. The major differences in training characteristics between junior and senior athlete seasons were higher sport-specific volumes of LIT and MIT and less HIT. These differences were accompanied by more shooting training, particularly at rest, and in connection with LIT.

## Introduction

Biathlon is a Winter Olympic sport that combines cross-country (XC) skiing over undulating terrain in the skating style with rifle shooting in competitions lasting ∼20–50 min. During competitions, biathletes carry a ∼3.5 kg rifle while skiing and stop two to four times to perform a five-shot series of shooting in either the prone or standing position (1). Although the physiological and technical demands of biathlon are comparable to those of XC skiing (2), the additional demands of shooting directly after high-intensity exercise separate biathlon from most other endurance sports (1). Accordingly, success in biathlon requires a well-developed aerobic endurance capacity and skiing technique combined with rapid and accurate shooting performed under high physiological strain and mental pressure (1).

To reach elite to world-class levels in endurance sports, a successful long-term development process and a progressive increase in training volume are required to ensure sustainability and gradual performance development (3). In a recent case study, Schmitt et al. (4) reported the long-term development process of a world-class male biathlete and showed a 32% increase in the overall training volume (530–700 h·season^−1^) from the age of 21 to 31. On average, the training consisted of 86% low-intensity training (LIT), 4% moderate-intensity training (MIT), 4% high-intensity training (HIT), and 6% strength training across the annual cycles investigated. In comparison, an 80% increase in training volume (522–940 h·season^−1^) was reported in the most successful female XC skier of all time from the age of 20 to 35 (5). Furthermore, a 27% increase in endurance training volume (462–635 h·season^−1^) was observed in a world-class male Nordic combined athlete from the age of 19 to 23 (6). However, knowledge of the progression of endurance training when combined with a mentally challenging task such as shooting in biathlon is still limited, and no data on the long-term training characteristics of world-class female biathletes exist.

In a recent review, Laaksonen et al. (1) provided an overview of the recent advances and perspectives in Olympic biathlon. Although biathlon has developed substantially over the last decades with a corresponding increasing scientific interest, most of the available literature has its emphasis on its competitive demands and performance-determining factors (7–12). In contrast, literature on the related training characteristics of elite to world-class biathletes is rather sparse, compared with that on successful XC skiers (5, 13–15). While some of the available literature on XC skiers training is also relevant to biathlon, many features unique to biathlon have significant implications for the training performed. For example, carrying a rifle influences both the physiological responses and kinematic patterns of skiing (16, 17), and the interspersed periods of shooting lead to even more interval-based fluctuations in exercise intensity compared with XC skiing (1). Moreover, the requirement for rapid and accurate shooting in biathlon involving features such as optimal rifle stability and triggering behavior as well as a reduction of range time and shooting time has obvious implications for training, where biathletes must balance a complex interplay between both physical and shooting training.

There is a need for a better understanding of the training characteristics of biathletes and particularly the challenging balance and progression of physical- and shooting-specific training over time. In addition, previous research on elite endurance athletes has primarily focused on the athletes' senior seasons. In biathlon, no data on the differences between junior and elite-level senior training exist, where athletes are classified as juniors from the age of 17 until they transition to seniors at the age of 23. Therefore, the main aim of this case study was to investigate the long-term development of performance, physiological, and training characteristics in a world-class female biathlete, emphasizing differences between the junior and senior athlete seasons.

## Methods

### Participant

The participant (born in 1981) is a highly decorated female biathlete with 4 Olympic medals (two golds), 18 World Championship medals (eight golds), 28 individual World Cup wins, and 4 podiums of the overall International Biathlon Union (IBU) World Cup. The Norwegian Social Science Data Services approved the study, and the participant provided written informed consent to participate.

### Overall design

To provide a comprehensive understanding and detailed insight into the participant's long-term development process, the study was divided into two parts: (1) a retrospective description of the participant's long-term performance, physiological, and training characteristics across 17 seasons from the age 17 to 33 years (1997–2014) and (2) detailed comparisons between three annual cycles as a junior athlete (18–20 years) and three annual cycles (years of peak performances) as a senior athlete (31–33 years). The senior seasons were chosen based on the performance level and further confirmed by the participant. To provide an accurate comparison with senior seasons, the first three junior seasons where the participant started prioritizing biathlon as her main sport were chosen.

### Performance data

The participant's performance development was based on performance analyses, including World Cup competitions, Olympic Games, and World Championships, downloaded from results publicly available (18).

### Training monitoring

The participant recorded her day-to-day training in both handwritten diaries (ages 17–22) and Microsoft Office Excel sheets (>22 years old) developed by the Norwegian Biathlon Federation. Physical training recorded for each session included the total duration of each training form (endurance and strength training), exercise mode (skiing, roller skiing, running, cycling, etc.), and intensity (LIT, MIT, and HIT). Skiing/roller skiing in the skating style was classified as specific endurance training, skiing/roller skiing in the classical style as semi-specific endurance training, and all other exercise modes (running and cycling) as non-specific endurance training. Shooting training recorded for each session included the number of shots fired during rest, LIT, MIT, HIT, or competitions and time spent on dry fire training (shooting without ammunition).

To record her physical training, the participant used a combination of the *session goal* and *time in zone* referred to as the *modified session goal approach*, as reported by Sylta et al. (19). As a junior athlete, the endurance training intensity was mainly controlled by using a combination of heart rate (HR) and rating of perceived exertion (RPE) during sessions. As a senior athlete, measurements of blood lactate were introduced and used in addition to HR and RPE and particularly in connection with MIT sessions and at altitude training camps. The five-zone intensity scale developed by the Norwegian Top Sports Center (20) was used as a framework to control and log her endurance training intensity with some adjustments throughout her career. The overall physiological boundaries between the different intensity zones used by both junior and senior athletes were LIT [zones 1–2, <2 mmol·L^−1^ blood lactate, 60%–82% of maximal HR (HR_max_)], MIT (zone 3, 2–4 mmol·L^−1^ blood lactate, 82%–87% of HR_max_), and HIT (zones 4–5, including competitions, >4 mmol·L^−1^ blood lactate, >87% of HR_max_). However, as a senior athlete, the participant became more accurate in her intensity control and used smaller target zones for different training intensities. Her target intensities were LIT (0.8–1.2 mmol·L^−1^ blood lactate, 65%–75% HR_max_), MIT (2–3.2 mmol·L^−1^ blood lactate, 85%–90% of HR_max_), and HIT (>3.2 mmol·L^−1^ blood lactate, 90%–95% of HR_max_), respectively. In all physical training sessions, shooting time was not included. For MIT and HIT sessions performed as intervals, time in the respective intensity zones included time spent from the first interval to the end of the last interval, excluding breaks. Strength training was recorded from the start to the end of that specific part of the session, including breaks. Speed training was not included in the analyses due to the unsystematic reporting of some stages of the participant's career. The included information on speed training is, therefore, solely collected through interviews with the participant. The researchers systematically analyzed all training data session by session using the framework and periodization phases previously reported (5). The annual cycles were categorized into the phases *general preparation period one* (*GP1*, *weeks 18–30*), *general preparation period two* (*GP2*, *weeks 31–43*), *specific preparation period* (*SP*, *weeks 44–52*), and *competition period* (*CP*, *weeks 1–13*). *The 4-week transition period* (*weeks 14–17*) *between CP and GP1 was not included in the analyses of the different phases.* Shooting training was systematized by calculating the total number of shots distributed into the categories shots fired during rest, shots fired during LIT sessions, shots fired during MIT/HIT sessions and competitions, and time spent on dry fire training.

### Physiological testing

Starting at the age of 22 years, the participant underwent regular physiological testing at three different test centers using similar equipment. The same standardized test protocol with measurements of the oxygen uptake (VO_2_) and HR was used throughout her career with a minor modification from the age of 24. The protocol consisted of 3 × 5 min stages with 1 min breaks at fixed speeds (2.5 m/s from the age of 22 to 24 and 2.75 m/s from the age of 25 to 32) and increasing inclination (5°, 6°, and 7°, respectively) during treadmill roller ski skating. From 22 to 24 years of age, the protocol was performed one time in the G2 sub-technique (21), while from the age of 25, the protocol was performed two times (3 × 5 min in the G2 sub-technique followed by 3 × 5 min in the G3 sub-technique). In some cases, the protocol was followed by an incremental test to exhaustion to determine the maximal VO_2_ (VO_2max_), using a protocol with fixed speed (2.5 or 2.75 m/s) and increasing inclination by 0.5°–1° every minute until exhaustion. HR and respiratory recordings using open-circuit indirect calorimetry with a mixing chamber (Oxycon Pro, Jaeger GmbH, Hoechberg, Germany) were collected over the final minute of each stage.

### Interviews

To gather additional information, complete missing data, ensure compliance with the training diary commentaries, and verify the training intensity of different training sessions, three semi-structured interviews with the participant were conducted during the data analysis phase of the study. The interviews were conducted face-to-face and tape-recorded.

### Statistical analyses

All data from the investigated seasons (junior vs. senior athlete) and annual phases (GP1, GP2, SP, and CP) are presented as mean ± standard deviation. The assumption of normality was tested by using a Shapiro–Wilk test in addition to visual inspection of *Q*–*Q* plots and histograms. Variables with normal distribution were analyzed using a paired-sample *t*-test for junior vs. senior seasons. Otherwise, the Wilcoxon signed-rank test was used. All statistical tests were processed using IBM SPSS statistics version 24 software for Windows (SPSS Inc., Chicago, IL, USA) and Office Excel 2016 (Microsoft Corporation, Redmond, WA, USA).

## Results

### Long-term performance characteristics

#### Childhood and youth

During the interviews, the participant described an active childhood with long walks or ski touring, fishing, and hunting in the mountains. The participant started training for XC skiing at the age of 8 and biathlon at the age of 10. At the age of 12–14 years, a typical week in the winter for her and her brother (who also became a world-class biathlete) included XC skiing training on Tuesdays and Thursdays, biathlon training on Wednesdays, and traveling around for competitions on weekends. In the summer, the participant also engaged in athletics (800 m as her favorite discipline) and long sessions of road cycling or running in the mountains.

#### Junior athlete

The participant started at a top sports high school at the age of 17, where she specialized even more in XC skiing and biathlon. During her first junior years (age of 16–18), she competed in both biathlon and XC skiing but identified herself most as a XC skier. The participant performed at a high national level in both sports before she started to prioritize biathlon only at the age of 18. She participated in the biathlon Junior World Championship at both the ages of 19 (bronze in the sprint, 9th in the pursuit, and 13th in the individual event) and 20 (24th in the sprint, 38th in the pursuit, and 21st in the individual event).

#### Senior athlete

At age 21, the participant was selected for the Norwegian senior biathlon national team and participated in her first World Cup competition. However, this was the start of a 2-year period characterized by stagnation and a lack of performance development. The participant confirmed this during interviews, suggesting it was caused by frequent changes of coaches and corresponding training philosophies, which she uncritically performed without paying attention to her body's signals. This probably led to a state of underperformance (non-functional overreaching or overtraining syndrome), and she had to take a break from training and competitions to regain her balance. The participant decided to transfer to the national development team, where she was encouraged by her coach to take greater ownership of her training process. From this point, she took more responsibility for both the planning and adjustment of her training. During this time, the participant also discovered that she had to increase her prioritization of shooting-specific training.

**Figure 1:**
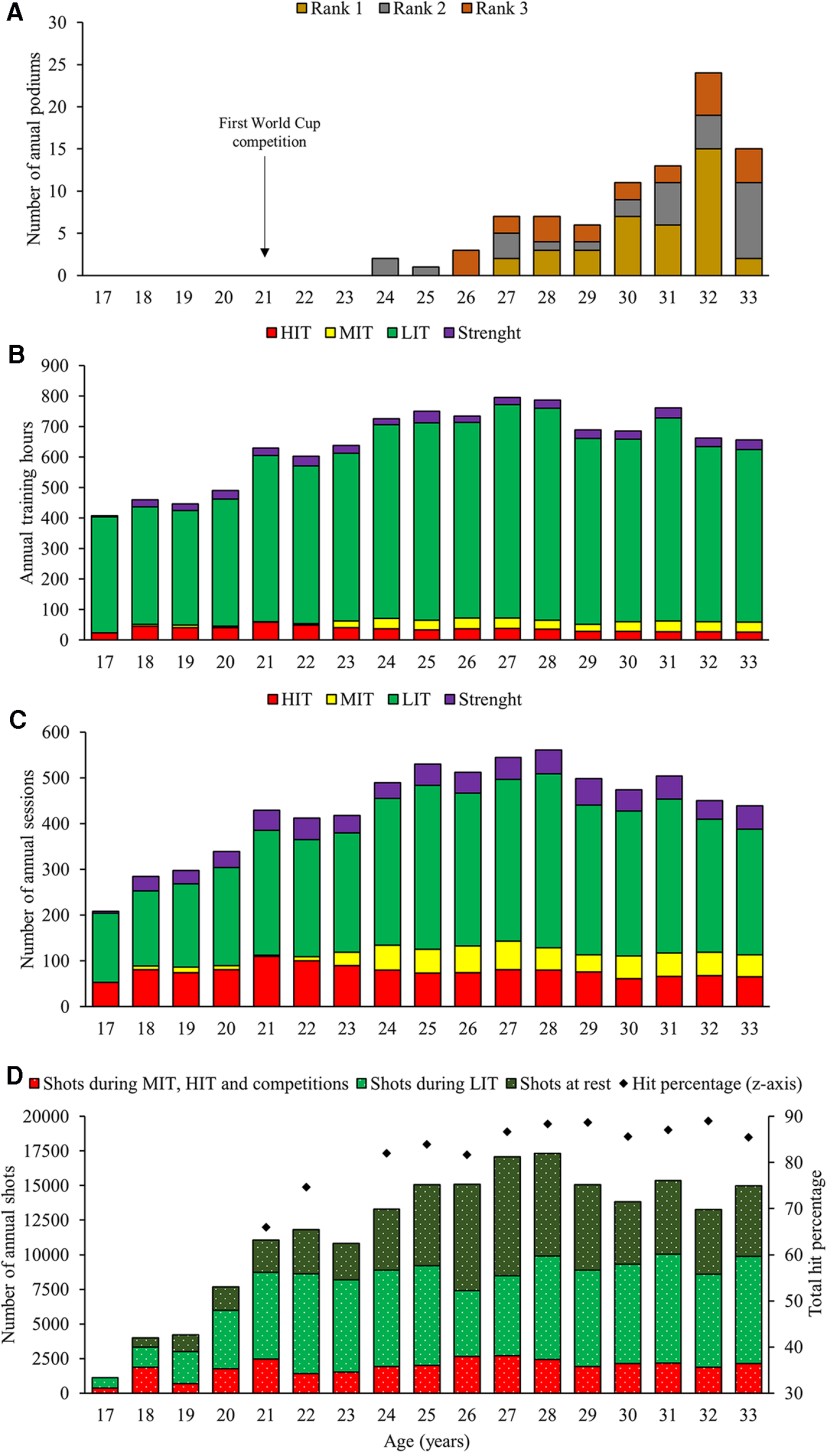
Annual top 3 performances in international competitions (**A**), the annual distribution of physical training distributed as endurance [low-intensity training (LIT), moderate-intensity training (MIT), and high-intensity training (HIT)] and strength training presented as training volumes (**B**), training sessions (**C**), and shooting training (shots at rest, shots during LIT, and shots during MIT, HIT, or competitions) and the total hit percentage of all international competitions (**D**) across 17 seasons in a world-class female biathlete.

In the subsequent season (age 24), the participant had her international breakthrough with two podiums in the World Cup. She achieved her first World Cup win at age 27 and her first Olympic gold medal at age 29. During the 3 years from 31 to 33 (defined as the seasons of peak performances), she was at the podium in the overall IBU World Cup (3rd, 1st, and 1st in 2011, 2012, and 2013, respectively) and won a total of 16 World Cup victories and 12 medals (eight golds) from international championships (World Championships and Olympic Games). The participant retired after winning three Olympic medals during her final season (age 33). The participant's performance development and associated training characteristics (physical and shooting) across the 17 seasons analyzed are presented in Figures 1A–C.

### Long-term physiological characteristics

**Figure 2:**
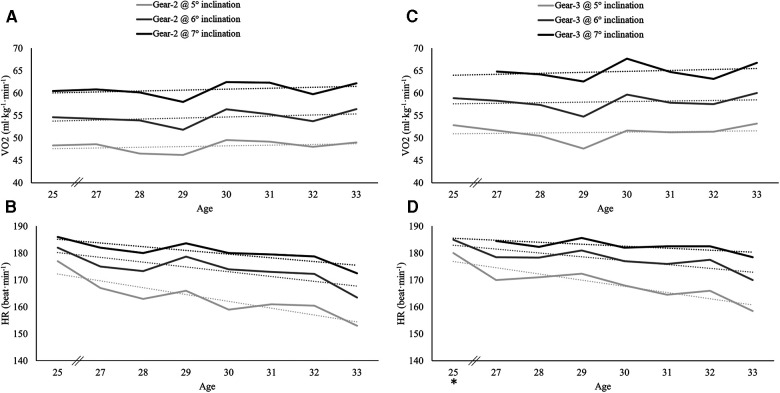
(**A–D**) Changes in oxygen uptake (VO_2_) and heart rate (HR) during roller ski skating from age 25 to age 33 in a world-class female biathlete. *The participant did not perform the G3 stage at 7° inclination at the age of 25.

The participant's body mass remained stable across the seasons including physiological testing (58.9 ± 0.5 kg). VO_2max_ in the G2 sub-technique was measured during GP2 in three of the seasons and increased from 62.9 ml·kg^−1^·min^−1^ at the age of 22 to 64.6 ml·kg^−1^·min^−1^ at the age of 23, and further to 69.2 ml·kg^−1^·min^−1^ at the age of 27. Moreover, there was an increase in VO_2_ and a corresponding decrease in HR in both the G2 and G3 sub-techniques over the 3 × 5 min stages performed from ages 25 to 32 (Figure 2).

### Long-term training characteristics

The annual volume of physical training increased by 95% (409–798 h·season^−1^) from the age of 17 to 27. This is a progression of 40 ± 50 h·season^−1^ and an increase from ∼8 to 15 h week^−1^. The training volume remained stable at the age of 28 before decreasing by 15% at the age of 29 and thereafter remained relatively stable (range of 657–763 h·season^−1^) from the age of 29 to 33 (Figure 1B). The annual number of shots fired increased substantially from the age of 17 to 28 (1,163–17,328 shots·season^−1^). On average, this constituted a progression of 1,473 ± 1,508 shots·season^−1^ and an increase from ∼22 to 333 shots week^−1^. From the age of 28 to 29, the annual number of shots fired decreased by 2,272 shots·season^−1^ before remaining relatively stable (range of 13,275–15,355 shots·season^−1^) from the age of 29 to 33 (Figure 1C).

### Comparison between junior and senior athlete seasons

#### Total training

Comparisons between three annual cycles as a junior athlete (18–20 years) and the three years of peak performances as a senior athlete (31–33 years) showed that the annual training volume was 48% higher as a senior than a junior (694 ± 60 vs. 468 ± 23 h·season^−1^, *P *= .030), while the number of sessions was 51% higher (464 ± 35 vs. 307 ± 28 sessions·season^−1^, *P *= .004). This included 50% more endurance training (662 ± 57 vs. 441 ± 19 h·season^−1^, *P *= .028) and 26% more strength training (31 ± 3 vs. 25 ± 3 h·season^−1^, *P *= .046). The volume of endurance training was 110% higher as a senior than a junior athlete in specific exercise modes (328 ± 30 vs. 155 ± 20 h·season^−1^, *P *= .026) and 71% higher in semi-specific exercise modes, although not significant (118 ± 42 vs. 69 ± 10 h·season^−1^, *P *= .245). In contrast, non-specific exercise modes remained relatively stable (216 ± 16 vs. 218 ± 24 h·season^−1^, *P *= .924). The proportion of specific/semi-specific/non-specific exercise modes was 50/18/33% as a senior athlete and 35/16/49% as a junior athlete. The weekly training patterns across the annual cycle are presented in Table 1.

**Table 1:** Weekly training distribution (mean ± SD) across the different periodization phases.

	Junior					Senior				
	Total	GP1	GP2	SP	CP	Total	GP1	GP2	SP	CP
Physical training										
Hours	8.9 ± 4.0	8.6 ± 4.1	10.2 ± 5.1	10.6 ± 3.6	8.4 ± 2.2	13.3 ± 6.2*	17.8 ± 3.9*	17.4 ± 3.6*	12.5 ± 4.5*	8.7 ± 4.0
Session	5.9 ± 2.5	5.5 ± 1.7	6.4 ± 2.8	6.8 ± 2.6	5.2 ± 2.2	8.9 ± 3.3*	9.5 ± 3.2*	8.7 ± 3.7*	9.4 ± 2.6*	9.4 ± 1.9*
Training forms										
Endurance (h·week^−1^)	8.4 ± 3.9	8.4 ± 3.9	9.6 ± 5.0	10.0 ± 3.7	8.0 ± 2.1	12.7 ± 5.9*	16.8 ± 3.7*	16.5 ± 3.3*	12.1 ± 4.3	8.5 ± 3.8
Strength (h·week^−1^)	0.5 ± 0.6	0.4 ± 0.5	0.6 ± 0.6	0.7 ± 0.5	0.4 ± 0.6	0.6 ± 0.6*	1.0 ± 0.5*	0.8 ± 0.5*	0.5 ± 0.3*	0.1 ± 0.3*
Exercise mode										
Specific (h·week^−1^)	3.0 ± 2.8	1.7 ± 1.5	2.0 ± 1.7	4.1 ± 3.4	5.4 ± 2.5	6.3 ± 2.9*	7.0 ± 2.3*	7.1 ± 1.9*	7.2 ± 2.6*	5.8 ± 2.7
Semi-specific (h·week^−1^)	1.3 ± 1.6	0.6 ± 1.0	1.1 ± 1.5	2.3 ± 1.8	1.7 ± 1.6	2.3 ± 2.0*	3.3 ± 1.9*	2.2 ± 1.6	2.4 ± 1.8	1.6 ± 2.0
Non-specific (h·week^−1^)	4.2 ± 3.5	6.1 ± 2.6	6.4 ± 3.6	3.6 ± 3.4	1.0 ± 0.9	4.2 ± 3.6	6.6 ± 2.7*	7.2 ± 3.2	2.5 ± 1.8*	1.1 ± 1.0
Specific/Semi-specific/Non-specific (%)	35/15/50	18/6/76	24/10/66	40/24/36	65/22/13	53/18/29	42/20/39	44/13/43	61/20/19	70/18/12
Intensity distribution										
LIT (h·week^−1^)	7.5 ± 3.6	7.3 ± 3.9	8.8 ± 4.6	8.9 ± 3.5	6.8 ± 1.7	11.6 ± 5.5*	15.5 ± 3.4*	15.1 ± 3.2*	10.8 ± 3.9	7.4 ± 3.4
MIT (h·week^−1^)	0.1 ± 0.3	0.3 ± 0.5	0.1 ± 0.3	0.2 ± 0.4	0.1 ± 0.2	0.6 ± 0.6*	0.9 ± 0.5*	1.0 ± 0.5*	0.6 ± 0.5*	0.3 ± 0.3*
HIT (h·week^−1^)	0.8 ± 0.8	0.8 ± 0.9	0.7 ± 0.5	0.9 ± 0.8	1.2 ± 0.8	0.5 ± 0.5*	0.4 ± 0.3*	0.4 ± 0.4	0.7 ± 0.5	0.8 ± 0.6
LIT/MIT/HIT (%)	89/1/10	85/3/12	91/1/8	89/2/9	85/1/14	91/5/4	92/6/2	91/6/3	89/5/6	87/3/10
Intensity distribution										
LIT (sessions·week^−1^)	3.6 ± 1.9	3.0 ± 1.1	4.1 ± 2.3	4.1 ± 2.0	3.1 ± 1.6	5.8 ± 2.5*	5.7 ± 2.2*	5.2 ± 2.8	6.4 ± 2.0*	6.7 ± 1.8*
MIT (sessions·week^−1^)	0.2 ± 0.4	0.2 ± 0.5	0.2 ± 0.4	0.3 ± 0.5	0.1 ± 0.3	1.0 ± 0.8*	0.8 ± 0.7*	1.0 ± 0.8*	1.2 ± 0.9*	1.0 ± 0.7*
HIT (sessions·week^−1^)	1.5 ± 1.2	1.8 ± 1.3	1.4 ± 1.2	1.4 ± 0.9	1.5 ± 1.4	1.3 ± 1.2	1.4 ± 1.3	1.2 ± 1.3	1.0 ± 1.1	1.5 ± 1.2
LIT/MIT/HIT (%)	69/3/28	62/4/35	72/2/26	70/5/25	70/2/28	73/12/16	73/10/17	71/13/15	74/15/12	72/11/16
Shooting training										
Total (shots·week^−1^)	101 ± 98	90 ± 114	153 ± 117	117 ± 79	102 ± 64	280 ± 177*	395 ± 182*	366 ± 144*	249 ± 108*	185 ± 101*
At rest (shots·week^−1^)	23 ± 49	39 ± 50	51 ± 73	11 ± 31	4 ± 15	97 ± 134*	223 ± 152*	138 ± 120*	25 ± 51	9 ± 34
During LIT (shots·week^−1^)	51 ± 62	39 ± 77	64 ± 61	73 ± 72	61 ± 55	143 ± 91*	135 ± 94*	183 ± 78*	178 ± 69*	131 ± 82*
During MIT, HIT, and competitions (shots·week^−1^)	27 ± 45	13 ± 26	38 ± 63	33 ± 51	38 ± 37	40 ± 28*	38 ± 28*	45 ± 25	46 ± 23	44 ± 29
Sessions with shooting										
Total (sessions·week^−1^)	1.7 ± 1.7	0.8 ± 1.1	1.9 ± 1.7	1.9 ± 1.5	2.6 ± 1.8	4.2 ± 2.2*	4.7 ± 2.0*	4.8 ± 1.7*	4.4 ± 1.6*	4.1 ± 2.2*
LIT shooting (sessions·week^−1^)	1.0 ± 1.1	0.6 ± 0.9	1.4 ± 1.2	1.2 ± 0.9	0.9 ± 0.9	2.1 ± 1.4*	2.7 ± 1.5*	2.7 ± 1.3*	2.0 ± 1.1*	1.5 ± 1.2*
MIT, HIT, and competitions (sessions·week^−1^)	0.8 ± 1.2	0.2 ± 0.5	0.5 ± 0.7	0.6 ± 0.9	1.7 ± 1.7	1.8 ± 1.3*	1.4 ± 0.9*	1.7 ± 1.0*	2.3 ± 1.1*	2.5 ± 1.6*
Dry fire training (sessions·week^−1^)	0.4 ± 0.8	0.4 ± 0.8	0.1 ± 0.2	1.0 ± 1.2	0.3 ± 0.7	3.2 ± 148*	3.3 ± 2.8*	4.4 ± 1.8*	3.1 ± 2.0*	2.9 ± 2.2*

GP1, general preparation period 1; GP2, general preparation period 2; SP, specific preparation period; CP, competition period; LIT, low-intensity training; MIT, moderate-intensity training; HIT, high-intensity training. *Significantly different from junior seasons (*P* < .05).

The annual volume of dry fire training (28.7 ± 9.4 vs. 12.2 ± 9.8 h·season^−1^, *P *= .043) and the number of sessions including shooting (216 ± 9 vs. 75 ± 14 sessions·season^−1^, *P *= .043) were higher as a senior than a junior. This difference in shooting-specific training included 175% more shots fired (14,537 ± 1,109 vs. 5,295 ± 3,425 shots·season^−1^, *P *= .016) in total and 321% more shots fired at rest (5,035 ± 321 vs. 1,197 ± 518 shots·season^−1^, *P *= .011) as a senior compared with a junior athlete. Further, the number of shots fired during LIT was 179% higher (7,440 ± 619 vs. 2,663 ± 1,975 shots·season^−1^, *P *= .031) and during MIT, HIT, and competitions was 44% higher (2,061 ± 174 vs. 1,435 ± 893 shots·season^−1^, *P *= .149) as a senior athlete.

#### Intensity distribution

**Figure 3:**
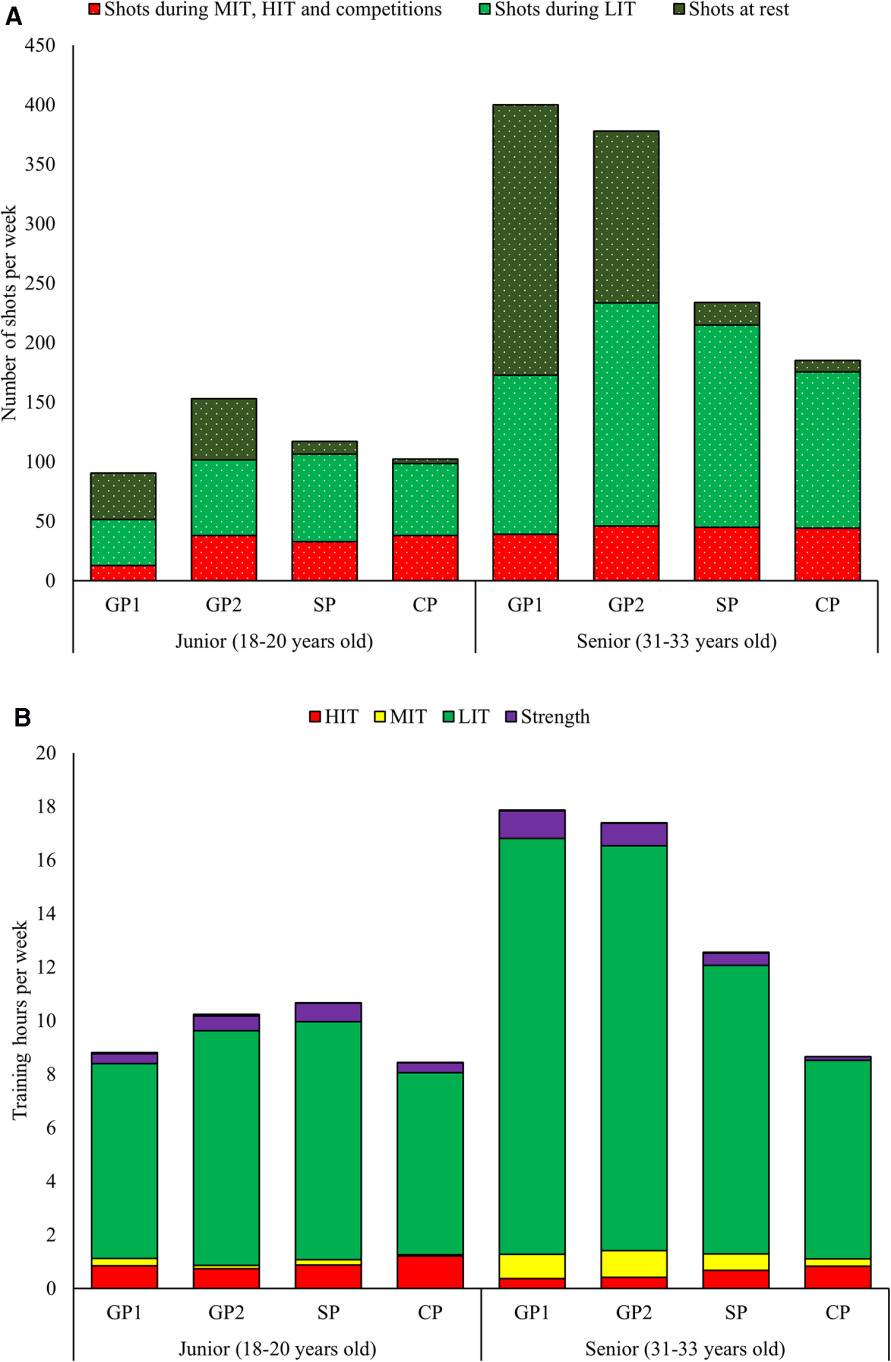
(**A,B**) Weekly distribution of shooting training (**A**) (shots at rest, shots during LIT, and shots during MIT, HIT, or competitions) and physical training (**B**) distributed as endurance [low-intensity training (LIT), moderate-intensity training (MIT), and high-intensity training (HIT)] and strength training across the annual phases in junior and senior seasons.

The annual distribution of endurance training intensity is presented in Figure 3. As a senior athlete, 91/5/4% of the annual endurance training time was distributed as LIT/MIT/HIT, respectively (73/12/15% in number of sessions). The corresponding distribution for junior athlete seasons was 89/1/10% for LIT/MIT/HIT, respectively (69/3/28% in number of sessions).

#### Annual periodization

The physical training volume was 124% higher in GP1, 97% higher in GP2, 35% higher in SP, and 4% higher in CP during the senior vs. junior seasons. Accordingly, the participant's periodization of physical training differed between the junior and senior seasons. While a traditional pattern with high training volumes in GP1 and GP2 followed by a clear reduction in training volume toward CP was observed in the senior seasons, an increase in training volume from GP1 to SP was observed as a junior. The intensity distribution also developed differently from GP1 to CP between the senior and junior seasons. In the senior seasons, a progressive reduction in both the volume and proportion of LIT and MIT with corresponding increased HIT was observed toward the CP. In contrast, the junior seasons included an increased volume of LIT from GP1 to SP before a reduction to CP, while HIT volumes remained relatively stable from GP1 to SP before increasing to CP (Table 1 and Figure 3).

The training volume in specific exercise modes was higher across all the annual phases in the senior vs. junior seasons. As a senior athlete, the proportion of specific exercise modes was relatively stable during GP1 and GP2 (42% and 44%, respectively) before increasing progressively to 70% in CP. As a junior athlete, the proportion of specific exercise modes was 18% during GP1 but increased progressively to 65% in CP. The proportion of semi-specific modes was relatively similar across all phases as senior vs. junior athlete, except for a higher proportion of semi-specific modes performed in GP1 as senior (20% vs. 6%).

**Figure 4:**
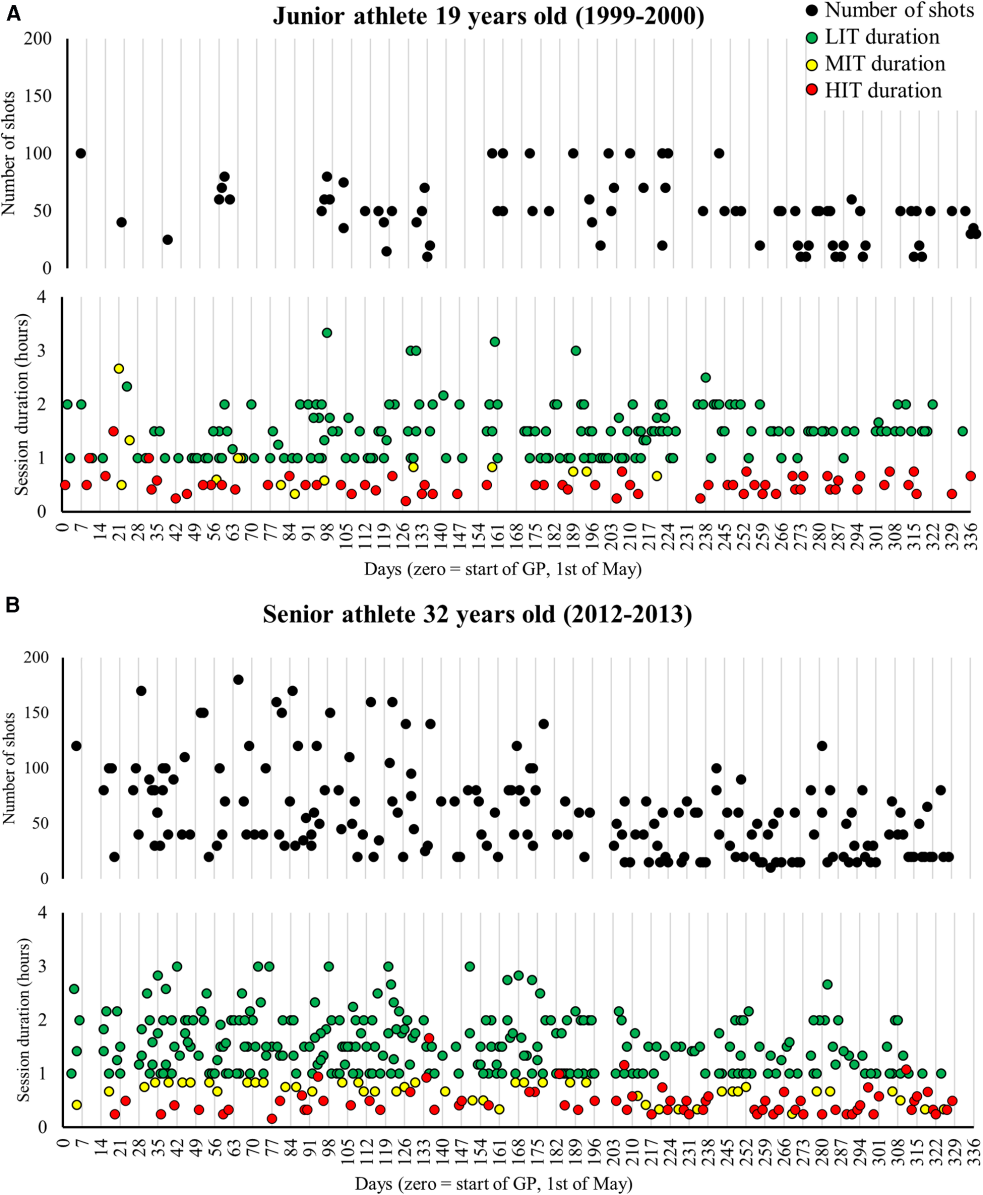
(**A,B**) The duration of low-intensity training (LIT), moderate-intensity training (MIT), and high-intensity training (HIT) sessions and number of shots fired across the annual cycle in one representative season as a junior (**A**) and a senior (**B**) athlete. *LIT sessions with a duration < 1 h are not included in the figures.

The number of shots fired as senior was substantially higher in the GP1 (564%) with somewhat smaller differences in the GP2 (147%), SP (100%), and CP (81%) compared with junior. As a senior, the overall number of shots fired followed the same pattern across the annual phases as the volume of physical training. As a junior, an increase in the number of shots fired was observed from GP1 to GP2, with a subsequent reduction toward the CP. The proportion of shots fired at different intensities across the annual phases was relatively similar between the junior and senior seasons. Therefore, large differences in the annual periodization of shooting training were observed in GP1, particularly caused by more shots fired at rest and LIT in the senior compared with junior athlete seasons (Figures 3, 4).

#### Low-intensity training

The annual LIT volume was 53% higher as a senior athlete compared with a junior athlete (602 ± 56 vs. 392 ± 22 23 h·season^−1^, *P *= .032), with a correspondingly higher number of sessions (301 ± 32 vs. 187 ± 25, sessions·season^−1^, *P *< .010). Furthermore, a higher number of annual sessions including shooting both in total (301 ± 32 vs. 187 ± 25, sessions·season^−1^, *P *< .010) and during LIT (86 ± 9 vs. 38 ± 20, sessions·season^−1^, *P *< .001) were performed as a senior compared with a junior. A typical LIT session as a senior vs. a junior included 8–16 vs. 8–12 series of 40–80 vs. 30–60 shots with a total duration of 1.5–2.5 vs. 1.0–1.75 h. Sessions without shooting were typically 1.5–3 vs. 1–2 h as a senior vs. a junior, respectively, using different exercise modes (running, roller skiing, or skiing) but could also be longer sessions of cycling (2–3 h) or hunting days (6–8 h walking in the mountains, typically registered as half of the time in the training diary). The distribution and duration of endurance training sessions at different intensities, as well as the number of shots fired in each session across one season and representative training weeks as a junior and a senior, are presented in Table 2 and Figure 4.

**Table 2:** Representative training weeks for the general preparation period 2 and the competition phase during junior and senior seasons.

Day/session		General preparation period 2		Competition period	
		Junior	Senior	Junior	Senior
Mon	1.	1 h roller ski bandy + 0.6 h strength training	0.5 h shooting at rest (90 shots) 2 h LIT roller ski skating w/shooting (70 shots)	1 h LIT running + 0.6 h general strength	1.25 h LIT ski classic
	2.		1 h roller skiing classic + 0.6 h strength training 10 min dry fire training		*Travel to World Cup destination*
Tue	1.	HIT session running with poles 0.5 h LIT warm-up 0.5 h HIT (2–3–4–4–3–2–1–2–1 min) 0.5 h LIT cool-down	2 h LIT roller ski skating	1 h LIT ski skating with/shooting (50 shots)	MIT session ski skating 0.5 h LIT warm-up (20 shots) 0.8 h MIT (3 × 6 min, 20 shots) 0.5 h LIT cool-down
	2.		0.5 h shooting at rest (100 shots) 1.5 h LIT cycling 10 min dry fire training		0.5 h LIT cycling + 0.25 h strength training 10 min dry fire training
Wed	1.	1.5 h LIT roller ski classic with/shooting (60 shots)	MIT session roller ski skating 0.5 h LIT warm-up (20 shots) 0.75 h MIT (5 × 8 min, 25 shots) 0.5 h LIT cool-down	1.25 h LIT ski classic	1.25 h LIT ski skating w/ shooting (50 shots)
	2.	1.5 h LIT running (undulating terrain)	1 h LIT roller ski classic 10 min dry fire training		10 min dry fire training
Thu	1.	0.5 h soccer + 0.6 h strength training	2 h LIT roller ski classic with shooting (70 shots)	*Travel to competition destination*	Competition 1 h LIT warm-up (20 shots) Competition (sprint) (20 min,10 shots)
	2.		0.5 h shooting at rest (100 shots) 1 h LIT cycling 10 min dry fire training		0.5 h LIT cycling 10 min dry fire training
Fri	1.	1.5 h LIT roller ski classic with shooting (80 shots)	2 h LIT roller ski skating with shooting and 8 × 15–20 s sprints (80 shots)	1.5 h LIT ski skating with 8 × 10–15 s sprints and shooting (50 shots)	1.25 h LIT ski classic with shooting (50 shots)
	2.		10 min dry fire training		10 min dry fire training
Sat	1.	HIT session roller ski skating 0.5 h LIT warm-up (30 shots) 0.4 h HIT test-race 6 km (20 shots) 0.5 h LIT cool-down	3 h LIT running/walking in the mountains	Competition 0.5 h LIT warm-up (15 shots) Competition (sprint) (20 min, 10 shots) 0.5 h LIT cool-down	Competition 1.1 h LIT warm-up (15 shots) Competition (relay) (20 min, 10 shots)
	2.				0.5 h LIT cycling 10 min dry fire training
Sun	1.	2 h LIT running/walking in the mountains	MIT session roller ski skating 0.5 h LIT warm-up (15 shots) 0.75 h MIT intervals (6 × 7 min, 35 shots) 0.5 h LIT cool-down	Competition 0.5 h LIT warm-up (15 shots) Competition (pursuit) (30 min, 20 shots) 0.5 h LIT cool-down	Competition 1.1 h LIT warm-up (15 shots) Competition (mass start) (30 min, 20 shots)
	2.		1.5 h LIT cycling 10 min dry fire training		*Travel to next World Cup destination* 10 min dry fire training
Total		Total volume (physical + shooting): 13.2 h Total volume (physical): 12.1 h LIT (hours/shots): 10 h/170 shots MIT (hours/shots): 0 h/0 shots HIT (hours/shots): 0.9 h/20 shots Strength and speed: 1.2 h Shooting at rest: 0 shots Dry fire training: 0 h	Total volume (physical + shooting): 24.5 h Total volume (physical): 21.1 h LIT (hours/shots): 18.6 h/255 shots MIT (hours/shots): 1.5 h/60 shots HIT (hours/shots): 0 h/0 shots Strength and speed: 1 h Shooting at rest: 290 shots Dry fire training: 1 h	Total volume (physical + shooting): 9.3 h Total volume (physical): 8.2 h LIT (hours/shots): 6.4 h/130 shots MIT (hours/shots): 0 h/0 shots HIT (hours/shots): 0.8 h (30 shots) Strength and speed: 1 h Shooting at rest: 0 shots Dry fire training: 0 h	Total volume (physical + shooting): 13.1 h Total volume (physical): 12.1 h LIT (hours/shots): 9.3 h/170 shots MIT (hours/shots): 0.3 h/20 shots HIT (hours/shots): 1.2 h/40 shots Strength and speed: 0.25 h Shooting at rest: 0 shots Dry fire training: 1 h

LIT, low-intensity training; MIT, moderate-intensity training; HIT, high-intensity training.

#### Moderate- and high-intensity training

The annual MIT volume was almost four times higher (34 ± 1 vs. 7 ± 2 h·season^−1^, *P *= .001), with a correspondingly higher number of sessions (50 ± 2 vs. 10 ± 2 sessions·season^−1^, *P *< .001) as a senior compared with a junior athlete. As a senior, a typical MIT session included a 0.5–1 h warm-up followed by 6–7 × 6–8 min intervals in a roller ski course (the course length decided the duration of each interval). The shooting was most often performed at the end of, although sometimes in the middle of, each interval. The proportion of MIT sessions including shooting increased toward the competition phase (GP1: 70%, GP2: 88%, SP: 89%, and CP: 94%). In the CP, MIT sessions had shorter duration and were typically performed as 3 × 5 min intervals (controlled intensity including 20 shots), for example, on Tuesdays when World Cup competitions were held on Thursday, Saturday, and Sunday. The participant mentioned during the interviews that she was very accurate with the intensity control during MIT sessions by consistently performing the first two intervals just below her target HR (85%–90% of HR_max_) and blood lactate (2.0–3.2 mmol·L^−1^) zones.

The annual HIT volume (including competitions) was 36% lower as a senior compared with a junior (27 ± 1 vs. 42 ± 3 h·season^−1^, *P *= .006), with a correspondingly lower number of sessions performed (66 ± 2 vs. 79 ± 4 sessions·season^−1^, *P *= .023). As a senior athlete, most HIT sessions were performed as 6 × 4 min, 8 × 3 min, or 5 × 5 min uphill running with poles. To ensure that she met the goal of the session, the participant rated her perceived training quality during all MIT and HIT sessions using a scale from 1 to 5. Furthermore, a simple rule of thumb was to have at least 1 day of LIT between MIT sessions and 2 days between HIT sessions. As a junior athlete, typical HIT sessions included test competitions in the skating style with shooting, or 4–8 × 2–4 min intervals, often performed as running or uphill running with poles. The participant also mentioned that she was not afraid of competing and took part in competitions both in running and orienteering in addition to XC skiing and biathlon during these years.

#### Strength and speed training

The annual strength training volume was 26% higher as a senior athlete compared with a junior athlete (31 ± 3 vs. 25 ± 3 h·season^−1^, *P *= .046), with a correspondingly lower number of sessions performed (47 ± 6 vs. 32 ± 3 sessions·season^−1^, *P *= .038). As a senior athlete, a typical strength session included 15–30 min of core/stabilization exercises followed by upper-body heavy strength training (3–4 series of 6–8 repetitions of maximal strength including 4–6 different exercises). As a junior athlete, a typical strength session included 30–45 min of various core/stabilization exercises targeting muscles involved in the force transfer during XC skiing. Speed training was unfortunately not included in the analyses, but the participant mentioned during interviews that she typically performed 6–8 × 15–20 s sprints across different terrains one to two times per week integrated as a part of LIT sessions. The content and frequency of these sessions were similar across the senior and junior athlete seasons.

## Discussion

This study investigated the long-term development of performance, physiological, and training characteristics in a world-class female biathlete, with emphasis on differences between junior and senior athlete seasons. The main findings were as follows: (1) There was a long-term progression in the annual physical training volume (∼409–792 h·season^−1^) and shots fired (∼1,163–17,328 shots·season^−1^) from the age of 17 to 28, with a subsequent reduction in both the physical training volume (694 ± 60 h·season^−1^) and shots fired (14,537 ± 1,109 shots·season^−1^) during the seasons of peak performance at ages 30–33; (2) VO_2max_ in roller ski skating increased by 10% (62.9–69.2 ml·kg^−1^·min^−1^) from age 22 to 32; (3) comparisons of junior vs. senior seasons demonstrated 48% higher physical training volumes and 175% more shots fired as senior, mainly due to more LIT and MIT with shooting and less HIT performed in the general preparation period.

### Long-term training characteristics

The participant followed a long-term progression in the annual volume of physical training (average increase of 40 h·season^−1^) before achieving her highest training volumes at the age of 27–28. These patterns are similar to those previously described in various world-class endurance athletes (4–6, 22–24), further supporting the importance of long-term progression in training volume to reach world-class endurance performances. Interestingly, novel data from this study showed that the progression of physical training coincided with an average increase of 1,200 shots per year, reaching a peak at the age of 27–28 (17,328 shots·season^−1^). However, a subsequent reduction to ∼13,275–15,355 annual shots during her seasons of peak performance (age of 31–33) was observed. While no previous data on the progression of shooting training is reported in the literature, the number of shots fired during the participant's seasons of peak performance is in line with the ∼12,000–15,000 annual shots previously reported in a world-class male biathlete (4). However, a substantially higher number of shots (∼22,000 shots·season^−1^) is reported in Swedish national team biathletes (1). Possible explanations for the observed differences in the number of annual shots fired might be individual variations in the requirement for shooting-specific training, differences in the quantification of shooting training (e.g., daily registration in a training diary vs. estimation by coaches), or further developments of the sport with increased demands for shooting-specific training after the participant retired from biathlon in 2014.

An interesting aspect of the participant's long-term development process was two seasons characterized by a lack of performance development from ages 21 to 23. Although at a later stage in their career, similar periods of stagnation have previously been described in two world-class XC skiers (25, 26). Some similarities in these athletes' return from underperformance include taking a break from systematic training and competitions, changing the training stimulus, and increasing their autonomy in the planning and adjustments of training. The participant also emphasized increased shooting-specific training as necessary to achieve her international breakthrough at the age of 24. However, contrary to the world's most successful female XC skier, whose most successful seasons coincided with the highest annual training volumes (5), the participant in this study reduced her volumes of both physical and shooting training during her seasons of peak performance. In line with this, the annual volumes of ∼650–750 h·season^−1^ physical training during these seasons are within the lower range of the training volumes (∼700–900 h·season^−1^) previously reported in world- or national-class biathletes (1, 4, 27, 28). The participant mentioned during interviews that the reductions in training volume were due to increased emphasis on improving the quality of each single training session and that this was particularly important to further develop her shooting skills. Consistent with these findings, a recent commentary highlighted the importance of training quality in endurance sports, and the quality of both the training process and the execution of each training session likely are important factors separating the highest-performing athletes from the rest (29). Although the participant's reduction in both the volume of physical and shooting training during her most successful seasons might seem contra intuitive, it likely contributed to increased load-recovery balance, training quality, and thereby better adaptations and performance development.

Furthermore, the participant's physical training volumes were ∼30% lower than the ∼900 annual training volumes reported for female world-class XC skiers (5, 14). Similar differences have previously been observed between national team XC skiers and biathletes (27, 28) and are likely explained by the additional demands for shooting-specific training in biathlon (1). Furthermore, the participant performed ∼20% higher annual training volumes in the skating style but less strength training (31 vs. ∼50–90 h·season^−1^) than previously reported in world-class XC skiers (2, 5). These findings indicate that biathletes likely compensate for lower physical training volumes than XC skiers by performing more specific training in the skating style. The reason for the lower strength training volume compared with XC skiing can only be speculated, and most likely, it reflects the participant's own prioritizations rather than differences in sport-specific demands between biathlon and XC skiing. Taken together, the observed differences in training characteristics between biathlon and XC skiing underpin the complex and demanding nature of biathlon, which requires an adequate load-recovery balance and training quality in both physical and shooting training.

### Long-term physiological characteristics

While several studies have reported VO_2max_ values in world-class endurance athletes (30), including biathletes and XC skiers during their most successful seasons (31), data on the long-term development of physiological capacities are relatively sparse. The participant increased her VO_2max_ in roller ski skating (G2 sub-technique) by 10% (∼63–69 ml·kg^−1^·min^−1^) from the age of 22 to 27. In comparison, increases of 4%–13% in VO_2max_ over a 7-year period have been reported in elite male rowers (23, 32). However, other studies including elite to world-class athletes have reported no long-term changes in VO_2max_ (22, 33, 34). Tønnessen et al. (31) reported average VO_2max_ values in running of 66 ml·kg^−1^·min^−1^ in female world-class biathletes, which were 10% lower than the corresponding values reported among distance XC skiers at the same performance level. Although the participant did not measure VO_2max_ during her most successful seasons, the highest VO_2_ values obtained during the incremental intervals in the G3 sub-technique were ∼67 ml·kg^−1^·min^−1^. Therefore, her VO_2max_ was likely to be at comparable values (68–70 ml·kg^−1^·min^−1^) with those previously reported in female world-class endurance athletes (5, 14, 22, 30, 31). Further, there was a clear reduction in HR during the incremental intervals throughout her career. These physiological changes likely had significant implications for her training and associated intensity zones, by allowing higher speeds and potentially better technical quality during LIT and MIT sessions. However, the use of different test centers, time points for testing, and lack of any direct measurements of VO_2max_ during the last seasons of her career indicate that the physiological data in this study should be interpreted with caution. By much of the same reasons, in addition to the use of different exercise modes (e.g., running vs. roller ski skating), comparisons of VO_2max_ values reported in previous studies should also be done with caution. Therefore, longitudinal data on the development of VO_2max_ and other physiological capacities are needed, both in biathletes and endurance athletes in general.

### Comparison between junior and senior athlete seasons

The annual physical training volume and number of sessions were ∼50% higher as a senior than junior athlete, with 175 % more shots fired. These increases in the volume of physical and shooting training from junior to senior athlete coincided with an increase in both the volume (155 to 328 h) and proportion (35% to 50%) of sport-specific training (i.e., skiing/roller skiing in the skating style). The intensity distribution of endurance training showed a transition from higher proportions of HIT as a junior athlete to higher proportions of LIT and MIT as a senior athlete. This was further confirmed by the participant during interviews, stating that the change in intensity distribution in part was due to changes in training philosophy and also a consequence of increased physiological capacities, making it possible to perform MIT sessions at higher and more competition-relevant speeds. Comparable changes in intensity distribution have previously been observed in the world's most successful female XC skier, emphasizing more HIT during the first part but more LIT and MIT during the latter part of her senior career (5, 35). However, similar intensity distributions from the age of 21 to 31 are reported in a world-class male biathlete (4). The most effective intensity distribution for endurance performance is widely debated, and while longitudinal data of endurance athletes often are characterized by increased LIT volumes, the progression and distribution of MIT and HIT are less clear (3, 36, 37). However, differences in the logging and quantification of endurance training intensity (i.e., *time in zone* vs. *session goal approach*) should be acknowledged in such interpretations (19, 36). For example, while the biathlete in the current study excluded the breaks during interval sessions, the world-class female XC skier included breaks in her logging of MIT and HIT sessions (5). Therefore, more long-term training data, following an accepted framework for quantification, would raise the possibility of comparing training characteristics across endurance athletes and sports and thus allow more valid comparisons adding considerable scientific and practical value.

The abovementioned differences in physical training were accompanied by large increases in both the volume and content of shooting training. Here, large differences between junior and senior athlete seasons were observed in the amount of dry fire training (12 vs. 29 h) and the number of shots fired at rest (1,197 vs. 5,035 shots·season^−1^) and during LIT sessions (2,663 vs. 7,440 h·season^−1^). As a senior athlete, shooting at rest was often performed as a session including 80–100 shots in the morning before LIT sessions, while 10 min of dry fire training typically was performed either before physical training sessions or in the evening. The participant mentioned that this training was important to fine-tune technical details connected to her shooting performance (e.g., shooting posture, triggering behavior, and rifle stability) and all movements related to reducing both the shooting and range time. This is further supported by previous studies suggesting that dry fire training is important to improve triggering behavior, rifle stability, and mental aspects of shooting (38, 39). Although the distribution of shooting-specific features as a senior athlete (36% at rest, 51% during LIT, and 24% at higher intensities) is in line with previous data in biathlon (1, 4), no comparable data for the differences in shooting training between junior and senior athletes exists. In this context, the 20-year period since our participant was a junior athlete should be acknowledged, and there is likely a need for more updated data on shooting training in both junior- and senior-level biathletes.

Substantial differences were observed in the annual training periodization between junior and senior athlete seasons. Large differences in training characteristics were found in GP1 and GP2, with almost twice the volume of physical training and five times more shots performed as a senior athlete compared with that as a junior athlete, with smaller differences observed in SP and CP. In line with previous observations (27), the senior seasons included a reduction in both the volume of physical training and shots fired at LIT and MIT, but increased HIT in sport-specific modes from GP1 to CP. In contrary, the junior seasons were characterized by an increase in both physical training volumes and shots fired, mainly caused by increased LIT, but with relatively similar amounts of HIT performed from GP1 to SP. The reasons explaining these findings are likely increased specialization and professionalism in biathlon as a senior athlete, allowing higher training volumes and more shots fired during GP1 and GP2, while more frequent traveling and competitions make it less possible to perform high training volumes in the CP. Altogether, our findings indicate that the transition from a junior to the senior elite level included a tolerance for higher sport-specific volumes of LIT and MIT (including shooting), but less HIT. In addition, an increased volume of shooting training besides the physical training (dry fire training and shooting at rest) particularly during the GP was a clear progression from a junior to a senior athlete.

#### Practical applications

The participant had an active childhood with relatively late specialization to biathlon at the age of 18, followed by a long-term annual progression in both the volume of physical and shooting training. The participant emphasized increased shooting training as necessary to reach her international breakthrough at the age of 24, where she also had a progressive change from emphasizing HIT to more MIT. Furthermore, the participant reduced both her volume of physical and shooting training during her most successful seasons with the intention of increasing the quality of each single training session. In comparison to XC skiers, biathletes seem to compensate for lower overall physical training volumes with higher volumes of sport-specific endurance training in the skating style. Although this study provides data on the sophisticated training characteristics of a world-class female biathlete, the limitations of a single-case approach should be considered in the interpretation of the present findings.

## Conclusions

This case study provides unique insights into the long-term development of physical and shooting training from the junior to the senior elite level in a world-class female biathlete. From the age of 17, the participant had a 10-year progression in both the annual volume of physical and shooting training accompanied by development of sport-specific physiological capacities. However, a reduction in both the volume of physical and shooting training was observed during the seasons of peak performance with the intention of increasing training quality. The major differences in training characteristics between junior and senior athlete seasons were higher sport-specific volumes of LIT and MIT and less HIT particularly during the general preparation period. These differences were accompanied by more shooting-specific training, particularly at rest, and in connection with LIT. More data on the training characteristics of larger samples of biathletes at different ages and performance levels are needed to further understand the complexity of long-term training and performance development of biathletes.

## Data Availability

The raw data supporting the conclusions of this article will be made available by the authors, without undue reservation.
